# Initial indicators for the prognosis of *Acinetobacter Baumannii* bacteremia in children

**DOI:** 10.1186/s12879-023-08639-5

**Published:** 2023-09-29

**Authors:** Yi Hong, Xiaochen Lin, Chunxu Zhang, Xingqiang Dong, Meihua Lu, Saihu Huang, Lili Huang, Chunmei Su, Zhenjiang Bai, Shuiyan Wu

**Affiliations:** 1 Pediatric Intensive Care Unit, Children Hospital of Soochow University, Suzhou, Jiangsu China; 2 Department of Pediatrics, Changshu Hospital, Nanjing University of Chinese Medicine, Suzhou, Jiangsu China; 3 Laboratory department, Children Hospital of Soochow University, Suzhou, Jiangsu China

**Keywords:** Children; *Acinetobacter baumannii*, Bacteremia, Mortality, Risk factors

## Abstract

**Background:**

Risk factors related to mortality due to *Acinetobacter baumannii* (AB) bacteremia have been unveiled previously, but early clinical manifestations of AB bacteremia based on prognosis remain uncovered.

**Methods:**

The demographic characteristics, clinical features, antibiotic susceptibility, and outcomes of 37 hospitalized children with laboratory-confirmed AB bacteremia from Suzhou, China, were collected and analyzed retrospectively.

**Results:**

Of the 37 children with AB bacteremia included in this study, 23 were males and 14 were females, with a median age of 4.83 (0.60 to 10.15) years. Among the children, 18 died (48.65%, 18/37) and 19 survived (51.35%, 19/37). The dead group had a significantly higher incidence of respiratory failure (p = 0.008), shock (P = 0.000), MODS (p = 0.000), neutropenia (< 1.5 × 10^9^/L) (p = 0.000) and serious neutropenia (< 0.5 × 10^9^/L) (p = 0.000) than those in the survival group. The death group had significantly more invasive procedures (2 or more) than that in the survival group at 2 weeks before onset (p = 0.005). The proportion of MDR-AB in the death group was significantly higher than that in the survival group (p = 0.000), while the PICS score was significantly lower in the survival group than that in the death group (p = 0.000). There was no significant difference in effective antibiotic use within 24 h between these two groups (p = 0.295). Among the 37 children with bloodstream infection of AB, 56.76% (21/37) of the underlying diseases were hematological diseases and oncology. Among them, 17 (81.00%) were died in the hospital. The proportion of white blood cells (p = 0.000), neutrophils (p = 0.042), eosinophils (p = 0.029), the ANC (p = 0.000) and lymphocyte (p = 0.000), the NLR(p = 0.011), hemoglobin (p = 0.001), platelets (p = 0.000), prealbumin (P = 0.000), LDH (p = 0.017), blood gas pH (p = 0.000), and serum potassium (p = 0.002) in the death group were significantly lower than those in the survival group. However, CRP (p = 0.000) and blood glucose(p = 0.036) were significantly higher in the death group than those in the survival group. By further multivariate analysis, CRP [OR (95% CI): 1.022(1.003, 1.041), p = 0.021] and neutropenia [OR (95% CI): 21.634 (2.05, 228.313, p = 0.011] within 24 h of infection were independent risk factors for death in children with AB bacteremia. When CRP was higher than 59.02 mg/L, the sensitivity of predicting mortality was 88.9%, and the specificity was 78.9%. And the sensitivity and specificity of neutropenia for predicting mortality were 83.3% and 84.2%.

**Conclusions:**

AB bacteremia has a high mortality in children, especially in patients with hematological diseases and oncology. Many early indicators were associated with poor prognosis, while elevated CRP and neutropenia were the independent predictors for the 30-day mortality of children with laboratory-confirmed AB bacteremia.

## Background

*Acinetobacter baumannii* (AB) was a nonfermented, oxidase-negative gram-negative bacterium that was considered an opportunistic pathogen that primarily infected nosocomial patients [1–3]. It has become an increasingly prevalent major bacteria for the nosocomial bloodstream infections (BSI), ventilator-associated pneumonia, and surgical site infections [4]. In children, primary disease, immunodeficiency, use of broad-spectrum antimicrobials and aggressive manipulation have led to an increase in the incidence of bloodstream infection. The resistant AB in bloodstream infection would lead to a skyrocketing increase of mortality and hospital costs [5].

Our hospital was a tertiary children’s hospital, and one of the characteristics of the intensive care unit of our hospital was that there are half patients with critical hematological tumors, and our clinical perception was that the early mortality is very high for hematological malignancy patients with *Acinetobacter baumannii*- bloodstream infection (AB-BSI). In adult patients with hematological tumors, the 7-day mortality was 72%, and the 30-day mortality was even as high as 96% [6], which was consistent with our clinical practice. Therefore, we hypothesized that for such an early high-mortality disease, the earlier the physician identified the risk factors, the more helpful it improved clinical outcomes.

Previous studies in adults and children have proposed previous exposure to carbapenems, hospitalization time of ICU, invasive procedures performed, severity of the disease, red blood cell distribution width, C-Reactive Protein (CRP) and neutropenia were risk factors for the mortality of AB-BSI [7–12]. Whether these clinically determined risk factors also have a certain hint at an early stage (24 h of onset), there is no answer until now. We also do not know the optimal cut-off value of these risk indicators. In order to provide early prognostic indicators for clinical practice, this study retrospectively analyzed the clinical data of children with AB-BSI within 24 h after the onset, aiming to find early warning indicators and the optimal cut-off of these indicators, so as to improve vigilance, active intervention, and ultimately reduce the mortality of children.

## Methods

### Clinical data collection

All children admitted to the pediatric intensive care unit of Children’s Hospital of Soochow University with the isolation of AB from blood were enrolled from January 2018 to December 2022. This study cohort comprised children aged 0.60-10.15 years.

Inclusion criteria of the patient was the following: (1) blood culture-confirmed AB bacteremia; (2) AB-BSI that occurred 48 h after admission, and met the diagnostic criteria for bloodstream infection; (3) on the day of specimen collection, the patient developed fever and had a temperature with more than 38.0 °C. Exclusion criteria was those including: (1) patients were detected with other pathogen from blood at the same time; (2) medical records was incomplete; (3) the length of hospital stay was less than 48 h.

Data of patients, including demographics, symptoms and signs, laboratory data, and empiric and definite antimicrobial agents within 24 h of the onset of the disease, and invasive manipulation within 2 weeks prior to infection were collected from the clinical records retrospectively. All patients were divided into survival and death groups according to their outcomes within 30 days. The study procedures were conducted in accordance with the declaration of Helsinki. Written informed consent was obtained from the family involved. The study was reviewed by the Institutional Review Board in the hospital (ethics approval number: 2020CS069).

### Strain isolation and drug susceptibility test

The blood was collected from children with suspected infections. Once the specimen was obtained, it was immediately sent to the microbiology laboratory. The bacteria was isolated and cultured according to the standard [13]. Bruker Bacteria Identification Mass Spectrometer (MALDI Biotyper, Bruker, Germany) was used for identifying the type of bacteria. Drug susceptibility was measured by the instrument (VITEK2 Com-pact, Mérieux, France) and the disc agar diffusion method to measure the minimum inhibitory concentration. The evaluation of drug susceptibility adopted the 2020 standard of the American Clinical Laboratory Standardization Committee [14].

### Definitions

Bloodstream infection (BSI) was defined as pathogens isolated from blood cultures with clinical manifestations consistent with sepsis syndrome [15]. Multidrug resistant (MDR) referred to the 2012 expert consensus on the diagnosis, treatment, prevention and control of AB infection in China [16]. Neutropenia was defined as absolute neutrophil count (ANC) lower than 1500/µL peripheral blood. Serious neutrophil was defined as neutrophil count lower than 500/µL. The neutrophil-to-lymphocyte ratio (NLR) was calculated as a simple ratio between the neutrophil and lymphocyte counts measured in peripheral blood.

### Statistical analysis

SPSS 26.0 statistical software was used for statistical processing. Continuous data was represented by the median and quartile interval [M (P25, P75)], and the Mann-Whitney U test was used for comparison between the two groups. The counting data was compared by the number of cases or percentiles, and the Fishers exact probability method was used for the comparison between groups. Multivariate Cox regression model was used to determine the independent risk factors for death of 30 days. To determine the association between factors and mortality, hazard ratios (HRs) and 95% confidence intervals (CIs) were estimated with adjustment of independent risk factors. P < 0.05 was determined as statistically significant.

## Results

### Demographics and clinical characteristics

Of the 37 children with AB-BSI included in this study, 23 were males and 14 were females, with a median age of 4.83 (0.60 to 10.15) years, and there were no significant differences in gender and age between these two groups. Among these patients, 18 died (48.65%, 18/37) and 19 survived (51.35%, 19/37). The dead group had a significantly higher incidence of respiratory failure (p = 0.008), shock (p = 0.000), MODS (p = 0.000), neutropenia (< 1.5 × 10^9^/L) (p = 0.000) and serious neutropenia (< 0.5 × 10^9^/L) (p = 0.000) than those in the survival group. In the 2 weeks before onset, 94.59% (35/37) of the children had 1 or more invasive procedures, of which 64.87% (24/37) had 2 or more invasive procedures. The children in the death group had significantly more invasive procedures (2 or more) than those in the survival group in 2 weeks before the onset of infection (p = 0.005). The number of people requiring endotracheal intubation (p = 0.045), deep vein catheterization (p = 0.000) and indwelling urinary catheters (p = 0.019) were significantly higher in the death group than that in the survival group, but there was no significant difference between the two groups in invasive procedures such as abdominal/thoracic drainage, PICC/PORT, CRRT, ECMO, and gastrointestinal endoscopy. The median length of hospital stay before onset was 22 (10, 36) days, and the median length of total hospital stay was 57 (33.5,105) days, of which 78.38% (29/37) had total hospital stay more than 30 days. The source of pathogen from ICU ward (including PICU, NICU, SICU) accounted for 86.49% (32/37), and that from general ward was 13.51% (5/37). The proportion of multidrug-resistant (also carbapenem-resistance) AB in the death group was significantly higher than that in the survival group (p = 0.000), while the PICS score was significantly lower in the survival group than that in the death group (p = 0.000). There was no significant difference in effective antibiotic use within 24 h between the two groups (p = 0.295) (Table 1).

**Table 1:** Clinical features between survival and death groups

	Death(n = 18)	Survival(n = 19)	P
Sex			
Male	13(72.22%)	10(52.63%)	0.313
Female	5(27.78%)	9(47.37%)	
Age(year)	5.54(3.31,10.74)	0.77(0.17,10.0)	0.066
Temperature(℃)	39.2(38.7,39.6)	38.5(38.2,39.5)	0.013
Complications			
Respiratory failure(n)	17(94.44%)	10(52.63%)	0.008
Shock(n)	17(94.44%)	3(15.79%)	0.000
MODS(n)	15(83.33%)	3(15.79%)	0.000
Bloody stool	4(22.22%)	1(5.26%)	0.180
Absolute neutrophil count			
<1.5 × 10^9^/L	17(80.95%)	1(6.25%)	0.000
<0.5 × 10^9^/L	14(66.67%)	0(0.00%)	0.000
Invasive manipulation (n)	3.5(2,4.25)	1(1,3)	0.001
Intubation/tracheostomy	14(77.78%)	8(42.11%)	0.045
Deep vein catheterization	16(88.88%)	5(26.32%)	0.000
Indwelling urinary catheters	7(38.89%)	1(5.26%)	0.019
Invasive manipulation ≧ 2(n)	16(88.88%)	8(42.11%)	0.005
Original from ICU(n)	18(100%)	14(73.68%)	0.046
Length of hospital stay before onset(d)	35(13,58)	21(11,88)	0.358
Length of hospital stay(d)	60(37,126)	39(22,65)	0.245
PCIS score	64(69,72)	86(80,90)	0.000
Multidrug-resistant bacteria(n)	17(94.44%)	6(31.58%)	0.000
Use of effective antibiotics within 24 h(n)	11(61.11%)	15(78.95%)	0.295

Note: Temperature, it is the highest temperature in the early 24 h of the onset of AB bacteremia

### Underlying disease

Among the 37 children with bloodstream infection of AB, 56.76% (21/37) patients with underlying diseases were haematological disorders and oncology, of which was consisted by 10.81% (4/37) acute lymphoblastic leukemia, 13.51% (5/37) acute myeloid leukemia, 2.70% (1/37) acute mixed cell leukemia, 5.41% (2/37) juvenile myelomonocytic leukemia, 8.11% (3/37) aplastic anemia, 10.81% (4/37) lymphoma and 5.41% (2/37) hemophagocytic lymphohistiocytosis. The rest included neonatal system diseases (16.22%, 6/37), circulatory diseases (8.10%, 3/37), digestive diseases (2.70%, 1/37), urinary diseases (2.70%, 1/37), endocrine diseases (2.70%, 1/37), nervous system (5.41%, 2/37), car accident (2.70%, 1/37) and immunosuppression after COVID-19 infection (2.70%, 1/37). Among the 21 children with haematological disorders and oncology, 17 (81.00%) were died in the hospital, of which 23.81 (5/21) were immunosuppressed post chemotherapy, 19.04% (4/21) were relapsed, and 57.14% (12/21) were the cases after bone marrow transplantation. However, only one child with non-hematological tumors (post-COVID-19 immunosuppression) died, and there was a significant difference in in-hospital mortality between hematological and non-hematological tumor patients. The number of children with hematologic diseases in the death group was significantly more than that in the survival group (94.44% vs. 21.05%, p = 0.000). But the number of children with neonatal systemic diseases in the survival group was significantly more than those in the death group (31.58% vs. 0%, p = 0.020) (Table 2).

**Table 2:** Underlying disease of these patients

Underlying disease(n)	Death(n = 18)	Survival(n = 19)	P
Hematological tumors	17(94.44%)	4(21.05%)	0.000
COVID-19 infection	1(5.56%)	0	1.000
Neonatal disorders	0	6(31.58%)	0.020
Circulatory disorders	0	3(15.79%)	0.230
Digestive disorders	0	1(5.26%)	1.000
Urinary disorders	0	1(5.26%)	1.000
Endocrine disorders	0	1(5.26%)	1.000
Neurological disorders	0	2(10.53%)	0.486
Car accident injuries	0	1(5.26%)	1.000

### Laboratory indicators within 24 h of infection

Among the 37 children with AB-BSI, the proportion of white blood cells (p = 0.000), the proportion of neutrophils (p = 0.042), the proportion of eosinophils (p = 0.029), the absolute neutrophil count (p = 0.000), the absolute lymphocyte (p = 0.000), the ratio of neutrophils to lymphocytes (p = 0.011), hemoglobin (p = 0.001), platelets (p = 0.000), and prealbumin (p = 0.000) LDH (p = 0.017), blood gas pH (p = 0.000), and serum potassium (p = 0.002) within 24 h of infection in the death group were significantly lower than those in the survival group. However, CRP (p = 0.000) and blood glucose (p = 0.036) were significantly higher in the death group than those in the survival group, and there were no significant differences in the remaining laboratory indicators (Table 3).

**Table 3:** Laboratory results of 37 children with Acinetobacter baumannii bloodstream infection

Index	Death(n = 18)	Survival(n = 19)	P
WBC(×10^9^/L)	0.29(0.1,1.5)	10.95(4.7,15.0)	0.000
N%	22.9(0.0,67.4)	58.20(43.1,73.5)	0.042
E%	0.1(0.0,1.0)	1.10(0.1,3.10)	0.029
L%	17.4(0.0,59.0)	32.3(15.9,42.6)	0.343
NLR	0.09(0,3.05)	1.6(0.9,4.4)	0.011
ANC(×10^9^/L)	0.05(0.0,1.1)	5.37(1.9,8.4)	0.000
L#(×10^9^/L)	0.13(0.0,0.4)	2.39(0.7,5.6)	0.000
Hb(g/L)	76.5(64.5,79.3)	98(84,119)	0.001
PLT(×10^9^/L)	36.5(8.3,70.0)	154(123,334)	0.000
CRP(mg/L)	163.4(91.8,200)	14.3(3.0,52.3)	0.000
PCT(ng/L)	6.6(0.6,34.8)	1.2(0.4,25.0)	0.394
Total protein(g/L)	50.4(45.4, 56.1)	59.5(51.8,67.4)	0.079
Albumin(g/L)	33.7(29.5,37.5)	38.1(33.0,41.0)	0.097
Globulin(g/L)	17.2(13.9,22.7)	21.7(16.7,26.1)	0.091
Prealbumin(mg/L)	92.00(58.5,114.5)	152(132,207)	0.000
AST(U/L)	27.5(16.1,96.3)	33.1(21.9,78.9)	0.743
ALT(U/L)	15.9(9.0,94.9)	22(14,52)	0.743
LDH(U/L)	206.5(157.8,387.7)	379.9(33.3,914.8)	0.017
Na+(mmol/L)	1371 ± 1.2	138.2 ± 1.0	0.480
K+(mmol/L)	3.3(2.6,3.8)	4.1(3.8,4.2)	0.002
Lactic(mmol/L)	1.9(1.4,3.4)	1.7(1.5,1.8)	0.159
G(mmol/L)	7.1(5.2,8.7)	5.8(5.1,6.6)	0.036
pH	7.2(7.1,7.4)	7.4(7.4,7.5)	0.000

### Analysis of risk factors for death

**Fig. 1:**
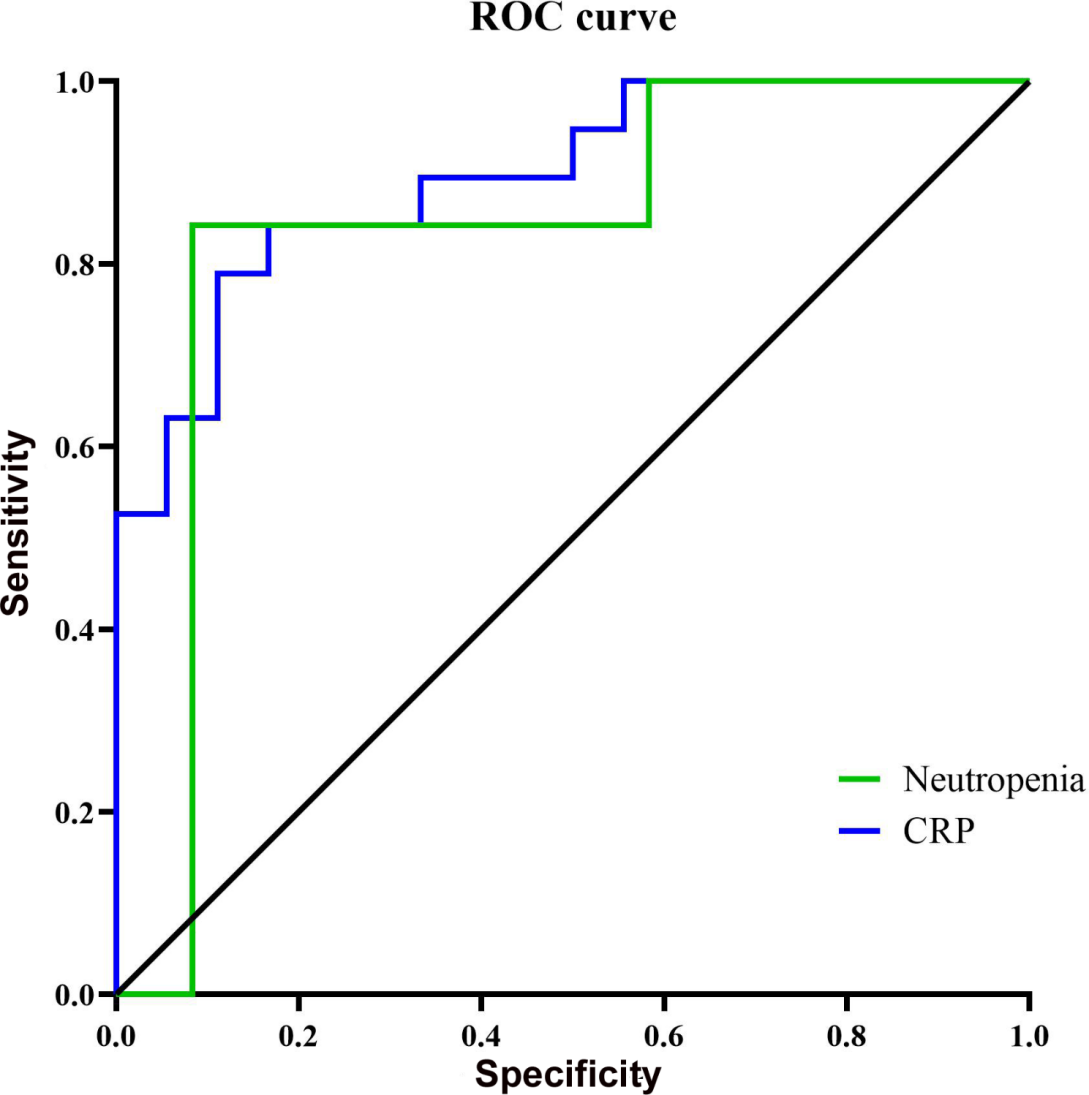
ROC curves was used to analyze the independent risk factors (CRP and neutropenia) for 30 days mortality

Due to the small sample size, we tried multivariate analysis for parameters with significant p-value and clinically determined risk factors for the mortality. Three univariates of statistically significant differences with CRP, reduced absolute neutrophil count and number of invasive operations (more than 2) were included in multivariate analysis. After COX regression correction, CRP (OR (95% CI): 1.022(1.003, 1.041), p = 0.021) and neutropenia (< 1.5 × 10^9^/L) (OR (95% CI): 21.634 (2.05, 228.313, p = 0.011) within 24 h of infection were the independent risk factor for death in children with AB-BSI (Table 4). When CRP was higher than 59.02 mg/L, the sensitivity of predicting mortality was 88.9%, and the specificity was 78.9%. While the sensitivity and specificity of the neutropenia (< 1.5 × 10^9^/L) for predicting mortality were 83.3% and 84.2%, respectively (Fig. 1).

**Table 4:** Multiple logistic regression analyse of early clinical manifestations potentially associated with 30d mortality

	OR(95%CI)	P value
CRP(mg/L)	1.022(1.003,1.041)	0.021
Invasive manipulation ≧ 2(n)	3.308(0.278,39.305)	0.343
ANC<1.5 × 10^9^/L(n)	21.634(2.05,228.313)	0.011

## Discussions

*Acinetobacter baumannii* (AB) was one of the six common multidrug-resistant bacteria “ESKAPE” (Enterococcus faecalis, Staphylococcus aureus, Klebsiella pneumoniae, *Acinetobacter baumannii*, Pseudomonas aeruginosa and Enterobacteriaceae) [17]. It could colonize in human and animal skin, wounds, respiratory tract and gastrointestinal tract, and was a more common opportunistic bacteria in clinical work [18]. An international cohort study showed that AB was one of the most common pathogens of hospital-borne bloodstream infection in intensive care unit, with severe organ dysfunction and even death [19]. The mortality had been reported to be between 29% and 73% [20, 21]. In our study, the mortality in children with AB-BSI was 48.65% (18/37). Therefore, it was of great significance to explore the early clinical characteristics and prognostic factors of children with AB-BSI, so as to optimize the prevention and treatment strategy of AB and reduce its mortality.

In this study, 94.59% (35/37) patients with AB-BSI had invasive procedures in the pre-stage (2 weeks before onset). Compared to the survival group, the death group had significantly more endotracheal intubation/tracheostomy, deep vein catheterization, and indwelling urinary catheters, which were risk factors for poor prognosis, as confirmed in many previous studies [22, 23]. AB has the ability to survive long periods in dry environments and on the surface of medical devices. These patients were basically from the ICU. They have been exposed to a variety of antibiotics in the early stage, and the immune system was damaged to varying degrees due to stress response. This condition made patients vulnerable to cross-transmission infections from healthcare workers or other patients after prolonged hospitalization, as well as from invasive procedures in which bacteria was prone to bacteremia if they enter the skin or mucosal barrier [24]. Ventilator-assisted ventilation and central venous catheters were both risk factors for bacteremia caused by multidrug-resistant AB, emphasizing the importance of strict standardized management of ventilators and central venous catheters in the ICU [25]。Indwelling catheterization was the most common risk factor for concurrent urinary tract infection (UTI), a catheter-associated UTI (CAUTI) that accounted for 35% of hospital-acquired infections [26] and often led to secondary bloodstream infections. Although recognition of this risk has led to a reduction in the insertion or the time of indwelling catheterization use, a significant number of hospitalized patients still need to undergo catheterization during their hospital stay. After CAUTI was diagnosed, the catheter should be removed (if possible) or replaced with a new catheter before starting antimicrobial therapy. Empiric antibiotic therapy was initiated in the early stage, but should be optimized later based on culture and susceptibility results later. Improper treatment of CAUTI was associated with the development of bacteremia and emergence of drug-resistant pathogen. Therefore appropriate CAUTI management was important to prevent the emergence of resistant pathogen, thus reducing poor outcomes and mortality [27]。Therefore, many measures such as standardized operation training of clinical medical staff, strict implementation of the aseptic process of invasive operation, and weighing the pros and cons of clinical decision-making to minimize and reduce invasive operation were of great value for reducing and shortening invasive operation as much as possible.

AB was a highly contagious and invasive opportunistic pathogen with high mortality and morbidity in immunodeficient patients [28]。Among the 37 children with AB-BSI, 56.76% (21/37) were children with hematological oncology, of which 23.81% (5/21) were immunosuppressed post chemotherapy, 19.04% (4/21) were relapsed, and 57.14% (12/21) were after bone marrow transplantation. Current treatments for hematological oncology included radiation therapy, chemotherapy, targeted therapy, CAR-T therapy, and hematopoietic stem cell transplantation. It was well known that these children with hematological oncology were in a state of acquired immune paralysis/deficiency and neutropenia. High-intensity chemotherapy and post-transplant immunosuppression led to mucosal damage, decreased immunity and neutropenia, greatly increased the risk of bloodstream infection. Moreover, multiple organ dysfunction and the exposure of broad-spectrum antibiotics before infection made the AB with MDR, thus the antibiotic options was narrowed. Once blood-borne infection occurred, it was easy to cause poor prognosis, and even life-threatening. Neonatal disorders accounted for 16.22% (6/37), of which 13.51% (5/37) were premature infants with respiratory failure, and 2.70% (1/37) were neonatal pneumonia. The imperfect development of the neonatal immune system and immature immune defense were important factors that cause bloodstream infection, so the incidence was higher among premature and low birth weight infants, but the mortality of AB-BSI in this cohort was not high as that in the patients of hematological oncology.

Moreover, it was also found that the white blood cells, hemoglobin and platelets in the dead group within 24 h of infection were significantly lower than those in the survival group. Among the 37 children with AB-BSI, 56.76% (21/37) had haematological disorders and oncology. The significant reduction of white blood cells, hemoglobin and platelets were closely related to the underlying diseases. As we all known, white blood cells and neutrophils can engulf and digest invading microorganisms and various necrotic cells, which was an important part of the body’s defense function. However, studies have shown that platelets, in addition to their traditional role in stopping bleeding, can directly identified, isolated and killed pathogens, regulated the behavior of white blood cells, and was one of the key regulator of intravascular immunity and inflammation in the host [29]. In this study, the reduction of ANC was an independent risk factor for poor prognosis in children with AB-BSI, which was consistent with the findings of Gu et al. [30] and Lee et al. [31]. We found that the sensitivity of neutropenia (ANC < 1.5 × 10^9^/L) within the first 24 h of infection for death was 83.3% and the specificity of that was 84.2%. After AB-BSI occurred, the innate cellular immune response was the first line to defend against pathogen invasion [32]. As the main innate immune cells to phagocytose and kill AB, neutrophils further transferred to the site of infection after macrophage infiltration, which was an important step to prevent the further aggravation of AB infection [33]. Neutrophils can dominated and eliminated AB through oxygen bursts and the formation of neutrophil extracellular traps [34].

It was well known that CRP level and PCT were associated with the severity of AB infection [35, 36]. In our study, we found that the CPR of the death group within 24 h of infection was significantly higher than that of the survival group, but there was no significant difference of PCT between the death group and survival group, which was consist with Feng’s study [36]. Additionally, multivariate analysis found that CRP was an independent risk factor for prognosis. But no one know which point would be the best cutoff of CRP for the prognosis of AB. And we further analyzed and found that the sensitivity of CRP (more than 59.02 mg/L) for death was 88.9% and the specificity for death was 78.9%. The conclusions provided more informations for the clinicans.

This study has some limitations. Firstly, there is an inevitable selection bias in the design of the retrospective study, and the influence of unmeasured variables and unknown confounding factors cannot be ruled out in this study. Secondly, the ability to distinguish deaths caused by AB-BSI from those caused by other factors, i.e., all-cause mortality rather than infection-related mortality in this study, is also a matter of concern in the future. Finally, the sample size of this single-center study was small, which may lead to lack certain generalization, and multicenter large sample studies are needed to further determine the prognostic factors of patients with AB-BSI.

In summary, AB bloodstream infection in childhood was prone to appear in immunosuppressed oncologic patients. Hematological diseases and oncology, organ dysfunction, decrease of white blood cells, neutrophils, lymphocytes, eosinophils, NLR, hemoglobin and platelets, elevated CRP, aggressive manipulation 2 weeks before infection and MDR were associated with poor prognosis. Moreover, elevated CRP (more than 59.02 mg/L) and neutropenia were the independent risk factors for 30-day mortality.

## Data Availability

The datasets used and/or analysed during the current study are available from the corresponding author on reasonable request.

## Abbreviations

AB: *Acinetobacter baumannii*
BSI: Bloodstream infection
MDR: Multidrug resistant
ANC: Absolute neutrophil count
NLR: Neutrophil-to-lymphocyte ratio
UTI: Urinary tract infection
